# Home environment relationships with children’s physical activity, sedentary time, and screen time by socioeconomic status

**DOI:** 10.1186/1479-5868-9-88

**Published:** 2012-07-26

**Authors:** Pooja S Tandon, Chuan Zhou, James F Sallis, Kelli L Cain, Lawrence D Frank, Brian E Saelens

**Affiliations:** 1 Seattle Children’s Research Institute, M/S CW8-6, P.O. Box 5371, Seattle, WA, 98145-5005, USA; 2 University of Washington, Seattle, WA, USA; 3 SDSU, San Diego, CA, USA; 4 University of California, San Diego, CA, USA; 5 University of British Columbia, Vancouver, Canada

**Keywords:** Social epidemiology, Childhood obesity, Children, Ecological models, Family

## Abstract

**Background:**

Children in households of lower socioeconomic status (SES) are more likely to be overweight/obese. We aimed to determine if home physical activity (PA) environments differed by SES and to explore home environment mediators of the relation of family SES to children’s PA and sedentary behavior.

**Methods:**

Participants were 715 children aged 6 to 11 from the Neighborhood Impact on Kids (NIK) Study. Household SES was examined using highest educational attainment and income. Home environment was measured by parent report on a survey. Outcomes were child’s accelerometer-measured PA and parent-reported screen time. Mediation analyses were conducted for home environment factors that varied by SES.

**Results:**

Children from lower income households had greater media access in their bedrooms (TV 52% vs. 14%, DVD player 39% vs. 14%, video games 21% vs. 9%) but lower access to portable play equipment (bikes 85% vs. 98%, jump ropes 69% vs. 83%) compared to higher income children. Lower SES families had more restrictive rules about PA (2.5 vs. 2.0). Across SES, children watched TV/DVDs with parents/siblings more often than they engaged in PA with them. Parents of lower SES watched TV/DVDs with their children more often (3.1 vs. 2.5 days/week). Neither total daily and home-based MVPA nor sedentary time differed by SES. Children’s daily screen time varied from 1.7 hours/day in high SES to 2.4 in low SES families. Media in the bedroom was related to screen time, and screen time with parents was a mediator of the SES--screen time relationship.

**Conclusions:**

Lower SES home environments provided more opportunities for sedentary behavior and fewer for PA. Removing electronic media from children’s bedrooms has the potential to reduce disparities in chronic disease risk.

## Background

Lower socioeconomic status (SES) has been consistently associated with poorer health in childhood [1,2]. Childhood socioeconomic circumstances also shape adult disease risks and explain in part the origins of adult health disparities. As with many other aspects of health, children in lower SES households in the U.S. and other developed countries are more likely to be overweight or obese [3,4].

There are likely multiple factors that mediate the SES--weight status relationship, [5] and it is important to identify modifiable factors that would improve children’s weight-related health behaviors. Children’s health behaviors develop within an ecological niche, [6] with the family environment being a critical influence. Factors such as access to media, parenting practices (e.g., rules about media), sibling influences, and family habits, may be important influences on children’s sedentary and active behaviors [7]. These home environment characteristics may be influenced by the parents’ educational attainment or income and in turn contribute to differences in children’s sedentary behavior, physical activity and ultimately, weight status.

Except for children’s screen time, which is negatively associated with SES, [8] family SES has generally not been found to be related to children’s activity levels, but further investigation examining parent education and income levels as separate measures is needed [9]. The present study aimed: 1) to determine if home activity environments differed by parental education and income levels and 2) to explore the processes by which the home activity environment mediates the association of family SES on children’s physical activity and sedentary behavior. It was hypothesized that the home physical environment would be less healthful in families of lower SES, and these families would have less active and more sedentary children.

## Methods

### Participants

Participants were part of the Neighborhood Impact on Kids (NIK) Study, an NIH funded longitudinal, observational cohort study of children aged 6 to 11 and a parent in Seattle/King County, WA and San Diego County, CA [10,11]. NIK was designed to evaluate the association of neighborhood and home environmental factors with children and parent’s weight status and weight-related behaviors. This study was approved by the Institutional Review Boards at Seattle Children’s Hospital and San Diego State University.

### Protocol

Participants were recruited September 2007 - September 2008 in San Diego and November 2007- January 2009 in King County. We attempted to contact a total of 8,616 households, of which 4,975 were screened for interest and eligibility, and 944 agreed to participate. Among families agreeing to participate, 730 consented and were enrolled. The final sample consisted of 713 child–parent pairs who completed the survey and had valid accelerometer data. Additional details regarding recruitment and inclusion/exclusion criteria have been previously published [10].

At a home or clinic visit, parents provided consent and children provided assent. The parent completed a survey (online or paper) that assessed, among other things, access to media and physical activity equipment at home, children’s sedentary behaviors, household rules and practices about physical activity and sedentary behavior, and sociodemographic information. The complete NIK survey is available at: http://www.seattlechildrens.org/research/child-health-behavior-and-development/saelens-lab/measures-and-protocols. Children and parents were instructed on having the child wear an Actigraph accelerometer for 7 days and were provided a log for recording when the accelerometer was worn. Study staff called participants several times within the week to answer questions and encourage daily wearing of the accelerometer.

### Measures

The highest level of reported education of the parent(s) in the household and the household income were both used as SES indicators. The original 7 categories for educational attainment (ranging from < 7^th^ grade to completed graduate/professional degree) and 11 categories for income (ranging from < $10,000 to > $100,000) on the survey were combined into 3 categories each for analyses according to the following *a priori* criteria: Education- low (≤completed high school), medium (completed college), high (completed graduate degree); income - low (≤$39,000), medium ($40,000-$89,000), high (≥$90,000). The Spearman’s rank correlation between household income and highest education in the household was 0.39. The 2008 median family income was $87,903 in Seattle/King County and $74,593 in San Diego County [12].

The physical home environment was assessed using survey items on the presence of electronic media in the child’s bedroom and access to fixed and portable equipment in and around the home that could be used for physical activity [13]. A Bedroom Media Score was generated using 5 items from a reliable scale which asks if a TV, DVD/VCR, computer, video game system and/or hand held video game player are present in the child’s bedroom (prior test-retest reliability ICC = .51 - .96) [14]. A Fixed Play Equipment Score was generated by summing yes/no items regarding presence of a basketball hoop, a swimming pool and/or a fixed swing set (prior test-retest reliability ICC = .53-.80) [14]. A Portable Play Equipment Score was generated based on access to a bike, jump rope, sports equipment (balls, racquets) and/or roller skates(prior test-retest reliability ICC = .60 - .82) [14].

Other home environment measures included the presence of parental rules on outdoor play and on media use, and parent, sibling and friend participation in sedentary and physical activities with the child. The Safety Rules Score was the sum of “yes” responses by parents about whether they had the following rules: “Stay close/within sight of house/parent,” “do not go into street,” “do not ride bike on street.”(prior test-retest reliability ICC = .61-.74) [14]. A Media Rules Score was generated by summing “yes” responses to the following 2 rules: “no TV before homework” and “<2 hours of TV per day” (prior test-retest ICCs of .57 and .73, respectively).

### Screen time, sedentary time and physical activity

Parents reported their children’s “typical weekday time” spent watching TV/DVDS, playing video games and using the internet/other electronic media with response options of none, 15 min, 30 min, 1 hr, 2 hrs, 3 hrs, ≥4 hours per day (prior test-tetest reliability ICC = .66, .73, .72 respectively). Responses were summed to create a parent-reported child screen time value in average hours/day.

Child overall physical activity and sedentary behavior were measured by the GT1M Actigraph accelerometer (Pensacola, FL). The Actigraph has been validated and calibrated for use among children [15]. Accelerometer data were collected in 30 second epochs. Participants were asked to wear the accelerometer for seven days during all waking hours. Upon return, the Actigraph was immediately downloaded and screened for completeness and irregularities/malfunction. A valid day was defined as having at least 10 valid hours of wearing time; and a valid hour contained no more than 20 minutes of consecutive zero counts. Data were included for children with at least 3 valid days. Data were converted to minutes engaged in sedentary behavior (≤ 100 counts per minute) and moderate-to-vigorous intensity physical activity (MVPA; ≥3 metabolic equivalents (METs)) using Freedson age-specific cut-points with the participant’s age rounded to half a year [16]. Data were also examined using Evenson (4 METs) cut-points given a recent study which found that these gave the best classification accuracy for all four levels of physical activity intensity and performed well among children of all ages [17,18]. Accelerometer data were cleaned and scored using MeterPlus version 4.0 (Santech, Inc., http://www.meterplussoftware.com).

As participants had been instructed to remove the accelerometers overnight, all data files were screened for non-zero counts between the hours of 11 pm and 6 am. In all, 93 participants were identified as having overnight activity during valid days. Participants' accelerometer log data were triangulated with the activity counts to determine sleep hours. In 92% of cases, the log-reported sleep start time corresponded with a significant drop in activity counts (below 1000), and the reported wake time corresponded to an increase in activity counts (above 1000) exactly or within 1/2-1 hour. In cases with the slight discrepancies between the log-reported times and activity counts, we relied more on the meter data and assigned the exact sleep and wake up times based on changes in activity counts. In 7 cases (including 3 sleepovers), there were discrepancies > 1 hour between the log and activity counts in the accelerometer data. In these cases, the data were reviewed by at least 2 individuals to arrive at an agreement about sleep hours based on activity counts (increase above 1000 or decrease below 1000). In 6 cases there was no log available and the following criteria were used: “asleep time” when counts dropped below 1000 for at least 3 consecutive 30 min blocks (1.5 hours) and “awake time” when counts increased above 1000 for at least 3 consecutive 30 min blocks (1.5 hours). Estimated sleep hours were converted to “non-wearing time” to prevent an overestimation of sedentary time due to the inclusion of overnight time.

Average accelerometer wear time for the whole sample was 5688 minutes. The differences in wear time across SES groups were not statistically significant. Wear times (in minutes) across income groups: Low: 5787, Med: 5750, High: 5640; across educational attainment groups: Low: 5772, Med:5675, High: 5622*.* Forty-seven percent of children’s accelerometer wearing time was spent at home.

Parents completed a place log of where their child went while wearing the accelerometer. Place categories were created to assess where children were while wearing the accelerometer. Accelerometer data were matched by day and time to the place log. From this, non-wear, sedentary, light, moderate, hard, and very hard accelerometer wear times were aggregated within the given timeframe of each location. For the purpose of the current study, the “Home” category included one single location for each participant (i.e. each child had only one address designated as home). If parent listed ‘front yard’ or ‘backyard’ in the place log, this was also considered *home*. “Home” did not include other parent/guardians’ homes or homes of relatives, friends or neighbors.

### Analysis

Children’s home physical activity and sedentary environments and their total and home-based activity levels were compared across different education and household income groups using chi-square test for categorical variables and linear regression for continuous variables. Parent’s age, marital status and ethnicity were included as covariates in the regression models as they differed across categories of income and education.

Home environment variables, which were found to vary in a statistically significant manner across SES, were selected for further analysis using the Sobel-Goodman test which tests if the indirect relationship between the independent and dependent variable through the mediator is significantly different from zero. Mediator analyses were conducted to examine the role of media in the bedroom, access to portable play equipment, rules around media and safety and parent screen time with their child as potential mediators in the statistically significant relationship between SES and screen time. For the mediation analyses, the original 11 categories of income and 7 categories of educational attainment were used (instead of the tertiles) in an effort to retain the most information. All analyses were conducted using STATA software version 10.1.

## Results

Parents were predominantly white, married, mothers with approximately half working ≥15 hours/week outside the home. Parents in higher SES households (both education and income) were older and more were married compared to lower SES households. The racial and ethnic composition of parents across SES tertiles was similar except for significantly fewer Hispanic households with higher educational attainment or income levels. (Table 1).

**Table 1:** Participant characteristics

**Characteristic**	**Highest Education in Household**^**a**^				**Household Income**^**b**^			
***N***	***Low***	***Med***	***High***	***p***	***Low***	***Med***	***High***	***p***
	***N = 165***	***N = 279***	***N = 261***		***N = 67***	***N = 218***	***N = 428***	
***Child’s age (years), mean (SD)***	9.3 (1.6)	9.1 (1.6)	9.1 (1.6)	*NS*	9.2 (1.4)	9.1(1.6)	9.1 (1.6)	*NS*
***Child’s gender, N (% Female)***	81 (49%)	143 (51%)	123 (47%)	*NS*	35 (52%)	115 (53%)	202 (47%)	*NS*
***Parent’s age (years), mean (SD)***	39.6 (6.4)	41.4 (5.2)	42.9 (9.7)	**<.001**	38.3 (7.1)	41.2 (6.2)	42.2 (5.2)	**<.001**
***Parent’s gender, N (% Female)***	146 (89%)	241 (85%)	221 (84%)	*NS*	64 (96%)	184 (84%)	363 (86%)	*NS*
***Parent’s race/ethnicity, N (%)***								
*White*	144 (89%)	246 (90%)	229 (89%)	*NS*	54 (87%)	187 (86%)	382(91)	*NS*
*Black*	4 (2%)	6(2%)	6(2%)		4 (6%)	6 (3%)	6 (1%)	
*Asian/Pacific Is*	7 (4%)	12 (4%)	13 (5%)		4 (6%)	13 (6%)	15 (6%)	
*More than one*	4 (2%)	5(2%)	7 (3%)		0(0%)	6 (3%)	10 (2%)	
*Other*	3 (2%)	5 (2%)	3 (1%)		0 (0%)	6 (3%)	5 (1%)	
*Hispanic*	54 (33%)	25 (9%)	14 (5%)	**<.001**	31 (46%)	38 (17%)	25 (6%)	**<.001**
***Marital status, N (%)***								
*Married*	132 (80%)	264 (95%)	251 (96%)	**<.001**	35 (52%)	195 (90%)	417 (97%)	**<.001**
***Hours worked outside home per week, N (%)***								
*< 15*	75 (46%)	150 (54%)	113 (44%)	*NS*	40 (60%)	102 (47%)	197 (47%)	*NS*
*15*–*35*	39 (23%)	61 (22%)	58 (22%)		14 (21%)	53 (24%)	93 (22%)	
*36+*	51 (31%)	68 (24%)	90 (34%)		13 (19%)	63 (29%)	133 (31%)	

*missing data on 0–16 participants for some variables.

a Defined by highest educated adult in household.

b Defined by annual household income.

A higher percentage of children in lower SES households had a TV, a DVD/VCR player, and a video game system in their bedrooms compared to children of higher SES (Table 2). There was no significant difference by SES in whether children had a hand held video game player or computer in their bedroom. Most children had access to portable active play equipment at or around their homes, but this access was higher in families of higher SES. Fewer children had access to fixed play equipment around their home, but this did not differ across SES. Approximately half of the children across SES categories reported having active video games at home, with no SES-based differences.

**Table 2:** Characteristics of Home physical activity environment by education and income

***Characteristic***	***Education***				***Income***			
***Items in child’s bedroom***	***Low***	***Med***	***High***	***p***	***Low***	***Med***	***High***	***p***
***(% yes)***	***N=165***	***N=279***	***N=261***		***N=67***	***N=218***	***N=428***	
*TV*	45%	16%	11%	*<.001*	52%	25%	14%	*<.001*
*DVD/VCR*	34%	16%	12%	*<.001*	39%	21%	14%	*<.001*
*Computer*	16%	12%	13%	*NS*	10%	13%	14%	*NS*
*Video game system*	23%	9%	8%	*<.001*	21%	15%	9%	*.004*
*Hand held video game player*	57%	59%	52%	*NS*	46%	55%	58%	*NS*
* MEDIA SCORE(0-5)	1.7(1.4)	1.1(1.1)	1.0 (1.1)	*<.001*	1.7 (1.4)	1.3 (1.3)	1.1 (1.2)	*<.001*
***Play equipment available at or around home (% yes)***								
***PORTABLE***								
*Bike*	93%	97%	98%	*.025*	85%	95%	98%	*<.001*
*Jump rope*	75%	81%	83%	*NS*	69%	78%	83%	*.02*
*Sports equipment (balls/bats/etc)*	94%	97%	97%	*NS*	91%	95%	98%	*.002*
*Roller skates, skateboard, scooter*	82%	89%	83%	*NS*	78%	83%	87%	*NS*
*PORTABLE EQUIP SCORE (0-4)	3.4(0.8)	3.6(0.6)	3.6(0.6)	*.01*	3.2(1.0)	3.5(0.7)	3.7(0.6)	*<.001*
***FIXED***								
*Basketball hoop*	54%	60%	60%	*NS*	48%	56%	62%	*NS*
*Swimming pool*	52%	55%	47%	*NS*	55%	46%	53%	*NS*
*Fixed play equipment (court, pool)*	56%	64%	61%	*NS*	55%	60%	63%	*NS*
* FIXED EQUIP SCORE (0-3)	1.6 (1.0)	1.8(1.0)	1.7(1.0	*NS*	1.6(1.0)	1.6(0.9)	1.8(1.0)	*NS*
*ACTIVE VIDEO GAMES*	56%	46%	48%	*NS*	51%	47%	50%	*NS*
***Parent rules enforced (% yes)***								
***SAFETY RULES***								
*Stay close to house/parent*	86%	81%	82%	*NS*	90%	87%	80%	*.02*
*Do not go into street*	79%	68%	74%	*.029*	84%	77%	69%	*.01*
*Do not ride bike on street*	69%	57%	55%	*.008*	73%	61%	55%	*.02*
*Do not cross busy streets*	96%	91%	89%	*NS*	96%	90%	92%	*NS*
*SAFETY RULES SCORE (0-4)	2.4(0.9)	2.1(1.0)	2.1(1.0)	*.01*	2.5(0.8)	2.3(1.0)	2.0(1.0)	*<.001*
***MEDIA RULES***								
*No TV/computer before homework*	83%	72%	79%	*.035*	87%	71%	78%	*.02*
*<2 hours TV/computer per day*	70%	70%	76%	*NS*	78%	63%	76%	*.002*
*MEDIA RULES SCORE (0-2)	1.5(0.6)	1.4(0.7)	1.5(0.7)	*NS*	1.6(0.5)	1.3(0.8)	1.5(0.6)	*<.001*
**Screen time with (mean days/week, SD)**								
*Siblings*	3.5 (2.6)	3.5(2.6)	3.1(2.5)	*NS*	3.3(2.7)	3.5(2.7)	3.3(2.6)	*NS*
*Parent/caregiver*	2.9 (2.5)	2.8(2.5)	2.3(2.2)	*.02*	3.1(2.7)	3.0(2.5)	2.5(2.2)	*.02*
**How often does an adult in the household (mean days/week, SD)**								
Watch child playing sports/PA*	3.1 (2.4)	2.8(2.2)	2.6(2.0)	*NS*	2.9(2.5)	2.8(2.2)	2.8(2.1)	*NS*
Encourage sports/PA*	4.5(2.4)	4.6(2.3)	4.6(2.3)	*NS*	4.3(2.6)	4.4(2.4)	4.7(2.3)	*NS*
Provide transport to sports/PA*	2.7 (2.3)	2.8(2.1)	3.0(2.0)	*NS*	2.3(2.0)	2.8(2.2)	2.9(2.0)	*NS*
*Do sports/PA with child*	2.0(2.1)	2.0(1.8)	1.9(1.6)	*NS*	2.4(2.4)	2.0(1.8)	1.9(1.7)	*.05*
*PARENT SUPPORT	3.4(1.8)	3.4(1.8)	3.4(1.7)	*NS*	3.2(1.9)	3.3(1.8)	3.5(1.7)	*NS*
**How often do your child’s siblings/ friends do sports/PA with child (mean days/week, SD)**	3.2(2.4)	3.6(2.2)	3.3(2.2)	*NS*	3.7(2.6)	3.3(2.3)	3.4(2.2)	*NS*

Though outdoor play rules were common across SES categories (Table 2), “do not go into street” and “do not ride bike on street” rules were significantly more common in low SES families. Parents in the middle income category had fewer rules regarding media use compared to those in the lowest and highest income categories.

Parents of lower SES tended to watch TV/DVDs with their children more often than in families of higher SES. Across SES levels, children watched TV/DVDs with their parents and siblings more days per week, on average, than they did physical activities with them. There were no statistically significant differences in parental support of physical activities and sports between SES groups.

Accelerometer-measured total daily MVPA, MVPA at home, total daily sedentary time and sedentary time at home did not differ significantly between levels of household education or income using 3 METs Freedson critera. The total daily MVPA results for the Evensen cut-points were lower but also were not significantly different across SES. Parents’ reported average daily screen time for their children varied significantly by SES, ranging from 1.7 in the high SES to 2.4 hours/day in the low SES families. (Table 3).

**Table 3:** Child’s daily screen time, sedentary time and physical activity by education and income

***Outcome***	***Education***^***a***^				***Income***^***b***^			
	***Low***	***Med***	***High***	***p***^***c***^	***Low***	***Med***	***High***	***p***^***c***^
	***N = 165***	***N = 279***	***N = 261***		***N = 67***	***N = 218***	***N = 428***	
	**Mean(SD)**	**Mean(SD)**			**Mean(SD)**	**Mean(SD)**	**Mean(SD)**	
***Screen time (parent-reported, hours/day)***	2.4(1.4)	1.9(1.3)	1.7(1.4)	**<.001**	2.4(1.6)	2.2(1.4)	1.7(1.3)	**.004**
***Child’s total sedentary time (accelerometer-measured, min/day)***	397 (68)	393 (71)	397 (69)	*NS*	394 (68)	402(69)	393 (71)	*NS*
***Child’s sedentary time at home (accelerometer-measured, min/day)***	189 (69)	192 (72)	188 (69)	*NS*	186 (74)	200 (76)	184 (65)	*NS*
***Child’s total Evenson (4-MET) MVPA (accelerometer-measured, min/day)***	44 (20)	48 (23)	48 (20)	*NS*	42 (18)	43 (21)	49 (22)	*NS*
***Child’s total Freedson (3-MET) MVPA (accelerometer-measured, min/day)***	142 (51)	149 (57)	147 (51)	*NS*	138 (51)	143 (56)	150 (52)	*NS*
***Child’s total 3-MET MVPA at home (accelerometer-measured, min/day)***	61 (37)	64 (38)	61 (35)	*NS*	62 (40)	64 (40)	61 (34)	*NS*

a Defined by highest educated adult in household.

b Defined by annual household income.

c Adjusted for parent’s age, marital status and ethnicity.

The mediation analysis found that some of the relationship between SES and children’s screen time was mediated by media in the child’s bedroom, access to portable play equipment and screen time with parents. (Table 4) For example, the estimated direct effect of income on screen time was -.08, so for each unit increase in income (on the 11-point scale), there was a .08 hours decrease in daily screen time. With media in the bedroom as a potential mediator, the indirect effect of income on screen time was -.02, which is a significant mediation effect (p value < .001). The size of the total indirect path suggests that approximately 23% of the total association between income and screen time was mediated through the amount of electronic media present in the child’s bedroom. Household rules on safety and media use were not found to be mediators in our analysis.

**Table 4:** Potential mediation effect of various home environment variables on the relationship between SES and children’s screen time

**Independent variable**	**Potential mediator**	**Direct effect estimate**^**a**^	**Indirect effect estimate**^**b**^	**% of total effect that is mediated**^**c**^	**p-value**^**d**^
Income	Media in bedroom	-.08	-.02	23%	**<.001**
	Access to portable play equipment	-.09	-.01	8%	**.04**
	Household rules about media	-.09	-.01	7%	.36
	Household rules about safety	-.10	.01	−1%	.50
	Screen time with parents	-.09	-.01	13%	**.01**
Education	Media in bedroom	-.18	-.08	31%	**<.001**
	Access to portable play equipment	-.24	-.01	6%	.05
	Household rules about media	-.25	.01	-.1%	.99
	Household rules about safety	-.25	.01	-.6%	.73
	Screen time with parents	-.23	-.03	10%	**.03**

^**a**^ Refers to the estimate of the direct effect of income or education on screen time.

^**b**^ Refers to the estimate of income or education on screen time through the pathway with the potential mediator.

^**c**^ Refers to the percentage of the total effect of income or education on screen time that is mediated by the potential mediator.

^**d**^ Is the p-value from the Sobel mediation test which tests the significance of the indirect effect of the potential mediator.

## Discussion

Children’s home environments for physical activity and sedentary behavior varied by socioeconomic status. Children in lower SES households had significantly greater access to electronic media devices in their bedrooms but lower access to portable play equipment. Household rules around outdoor play were more restrictive in lower SES households. These differences were found across both household income and highest level of educational attainment in the household. Children’s screen time was higher in low-SES households but there were no SES differences in children’s overall or home-based MVPA or sedentary time.

The SES disparities in screen time are similar to previous studies that found inverse associations between SES and screen-based media use [19,20]. Approximately half of the children from low SES families in this sample had a television in their bedroom and a quarter had a video game system, significantly higher than for children from high SES families. This paradox between low SES and high access to often expensive equipment has been explained by findings suggesting that parents in low SES families have greater concerns about their neighborhood’s safety [21], may lack time to supervise children in their neighborhoods, [22] and have less access to alternative activities, [23,24] which makes indoor screen-based entertainment an appealing alternative to outdoor play. Extensive marketing of electronic entertainment devices may be another contributing factor. Furthermore, higher parental SES may be related to greater awareness of and ability to adopt screen time recommendations; supporting the theory that many initiatives intended to improve population health also may increase disparities since social position determines how well one can adopt preventive health knowledge [25]. Our screen time results (mean of 1.9 hours/day), however, were lower than national estimates that suggest children this age are exposed to over 3–4 hours of screen time per day [20].

The physical activity results in this study are consistent with those of a review which found that various estimates of family SES were generally unrelated to children’s physical activity [9]. Ferreria et al. hypothesized that since physical activity in younger children is mostly informal, it may not involve much extra financial cost. As activity levels generally decline with age and associated costs for athletic participation potentially increase, perhaps such disparities in income affect physical activity more in adolescents [26] and adults [27]. Of note, family support for physical activities (watching, encouraging and providing transport to sports/physical activities) did not differ by SES in this sample.

Though sedentary behavior may displace some physical activity, it is not simply the inverse of active behavior, and sedentary time is also independently associated with poor health outcomes [8,28]. Thus, focusing efforts on modifiable factors to both decrease sedentary behavior and increase physical activity in high risk groups is critical. Analyses identified some potentially modifiable factors in the home environment that were found to mediate the relationship between SES and screen time, a common sedentary behavior. Media in the bedroom, access to portable play equipment and joint screen time with parents are all potentially modifiable in interventions. Portable play equipment may stimulate active behaviors that are incompatible with screen time, though affordability of some equipment could be a challenge for low income families. More marketing of play equipment or counter-marketing to electronic entertainment targeted to low SES families may be required.

Joint media use has been recommended so parents can monitor their children’s television exposure, help children interpret what they see, and moderate the impact of media exposure by reducing adverse effects and increasing the possibility of benefit [29]. However, a previous study found that co-viewing was not motivated by parental determination to mediate children’s television experiences, and it occurred less often with younger children who need it most [30]. That study found parents co-view with children when their viewing preferences coincide, and co-viewing is associated with positive parental attitudes towards television. Thus, excessive parent–child joint screen time appears to be a risk factor for child screen time and is an under-studied correlate of child sedentary time that could be targeted in an intervention. A better understanding of how families spend time together and interventions that promote joint physical activities could be helpful.

Media in the bedroom, especially TV, may be the most important mediator identified here because it has been associated with overweight, likely for several reasons, including greater screen time, [31] interference with sleep, [32,33] and increased exposure to advertising for unhealthy foods. Previous research has found that media in the bedroom mediates the relationship between SES and BMI in adolescents [34]. Our study highlights the need to target media in the bedroom at even younger ages.

There are some study limitations that warrant consideration. First, our screen time outcome was by parent-report, which has been shown to correlate with actual viewing time, [35] but is still subject to social-desirability biases that may differ by SES. Second, we did not examine school and neighborhood level factors in this study, which likely vary by SES and contribute to overall physical activity and sedentary behaviors, as well as physical activity opportunities in the school and neighborhood. Third, given the cross-sectional data for this study, we were unable to evaluate causality. Fourth, there are multiple scoring decisions and sets of cut-points for accelerometer data in children (e.g. 3 METs vs. 4 METs for moderate activity), and results can change significantly if different criteria are used, making comparisons between studies difficult [36]. We focused on the 3 MET cut-point for moderate intensity physical activity because this is the level specified in the US physical activity guidelines, [37] but we did analyses using 4 MET Evenson criteria as well for comparison. Fifth, we developed a novel method (using all the information available to us) for handling accelerometer wear time during sleep hours in order to minimize inflation of sedentary time. However this approach has not been validated and we may have inadvertently eliminated some wear time. Sixth, as many complex factors influence children’s activity levels, unmeasured factors that are related to both SES and activity levels likely exist. Outdoor time, in particular, has been found to be correlated with physical activity in children and was not specifically measured in our study [9,38]. Seventh, our sample had relatively small numbers of families in the lower SES groups, and generally high levels of physical activity across SES, potentially limiting the generalizability of our findings.

Present findings are a step in understanding SES disparities in childhood obesity. The finding that low SES home environments have more electronic devices in bedrooms and fewer pieces of play equipment than in high SES homes is cause for concern. Sedentary-promoting devices in the bedroom emerged as an important mediator of the SES-sedentary behavior association. Additional research is recommended that can inform interventions to improve the healthfulness of home environments of low SES families.

## Abbreviations

BMI, Body mass index; NIK, Neighborhood Impact on Kids Study; SES, Socioeconomic status; PA, Physical activity; METs, Metabolic equivalents; MVPA, Moderate-to-vigorous physical activity.

## Competing interests

The authors declare that they have no competing interests.

## Authors’ contributions

PT and BS developed the study design for this manuscript. BS is the pi and js and lf are the co-investigators of the NIK study, from which data for this manuscript were obtained; BS, JS and LF designed the NIK study. Data analyses were conducted BY PT and CZ. PT wrote the initial draft of the manuscript and all authors critically reviewed and revised versions. All authors read and approved the final manuscript.
